# Supplementary material for: COVID-19 incidence and mortality in non-dialysis chronic kidney disease patients
Source: PLoS One. 2021 Jul 9;16(7):e0254525. doi: 10.1371/journal.pone.0254525 (PMC8270438; doi:10.1371/journal.pone.0254525)
Supplement: S1 Appendix — (DOCX) [file pone.0254525.s001.docx]

**Supporting Information to “COVID-19 incidence and mortality in non-dialysis chronic kidney disease patients”**

Dino Gibertoni^1*^¶, Chiara Reno^1^¶, Paola Rucci^1^, Maria Pia Fantini^1^, Andrea Buscaroli^2^, Giovanni Mosconi^3,4^, Angelo Rigotti^5^, Antonio Giudicissi^4^, Emanuele Mambelli^5^, Matteo Righini^2^, Loretta Zambianchi^3^, Antonio Santoro^6^, Francesca Bravi^7^, Mattia Altini^7^

^1^Department of Biomedical and Neuromotor Sciences, University of Bologna, Bologna, Italy

^2^ Unit of Nephrology and Dialysis, “Santa Maria delle Croci” Hospital, Ravenna, Italy

^3^ Unit of Nephrology and Dialysis, “Morgagni-Pierantoni” Hospital, Forlì, Italy

^4^ Unit of Nephrology and Dialysis, “M. Bufalini” Hospital, Cesena, Italy

^5^ Unit of Nephrology and Dialysis, “Infermi” Hospital, Rimini, Italy

^6^ Specialty School of Nephrology, University of Bologna, Bologna, Italy

^7^ Local Healthcare Authority of Romagna (AUSL Romagna), Ravenna, Italy

*Corresponding author:

e-mail: dino.gibertoni2@unibo.it

¶These authors equally contributed to this work.

**Table A: ICD9-CM codes used to identify comorbidities**

| Comorbidity | List of ICD9-CM codes |
| --- | --- |
| Cardiovascular comorbidities | 393-398.99, 410*, 411*, 412*, 413*, 414*, 421*, 422*, 425*, 426*, 427*, 428*, 429*, 430-434.99, 436-438.99, 440-448.99, 557*, 745*, V151, V422, V432, V433, V450 |
| COPD | 490-496 |
| Tumors | 140-208.9, V10* |
| Liver disease | 070.22, 070.23, 070.32, 070.33, 070.44, 070.54, 070.6 070.9, 570*, 571*, V42.7, 456.0-456.2, 572.2-572.8 |
| Diabetes | 250* |
| Dementia including Alzheimer’s disease | 290*, 293.0, 293.1, 293.9, 294.0, 294.1, 294.8, 294.9, 310*, 331.0, 331.2 |

Table A shows the Elixhauser codes used to identify comorbid conditions. Because dementia is not included as a specific diagnosis in the Elixhauser algorithm, we used a specific set of codes to characterize this condition.

**Table B. Characteristics of the study cohort by CKD-EPI stage**

|  | All patients (n=4716) | 1-2 (n=340, 7.2%) | 3a (n=1014, 21.5%) | 3b (n=1845, 39.1%) | 4 (n=1340, 28.4%) | 5 (n=176, 3.7%) | χ^2^ test; p-value |
| --- | --- | --- | --- | --- | --- | --- | --- |
| Age at 1.01.2020, mean±sd | 76.2±11.6 | 63.6±15.1 | 73.2±11.4 | 77.8±9.9 | 79.5±10.2 | 77.2±12.2 | 533.3; <0.001* (1-2<all; 3a<3b, 4, 5; 3b<4; 4>5) |
| Males, n(%) | 3086(65.4) | 248(72.9) | 817(80.6) | 1187(64.4) | 747(55.7) | 85(48.3) | 190.6; <0.001 |
| eGFR at last visit (ml/min/1.73m^2^), mean±sd | 38.1±15.8 | 74.4±15.5 | 51.2±4.1 | 37.2±4.3 | 23.6±4.1 | 11.7±2.5 | <0.001* |
| Hypertensive nephropathy, n(%) | 3274(69.4) | 158 (46.5) | 680 (67.1) | 1353(73.4) | 967(72.2) | 115(65.3) | 106.7; <0.001 |
| Diabetic nephropathy, n(%) | 432(9.2) | 44(12.9) | 84(8.3) | 173(9.4) | 117(8.7) | 14(7.9) | 7.5; 0.112 |
| Polycystic kidney, n(%) | 98(2.1) | 13(3.8) | 12(1.2) | 29(1.6) | 33(2.5) | 11(6.2) | 27.4; <0.001 |
| Pyelonephritis, n(%) | 229(4.9) | 25(7.3) | 58(5.7) | 73(4.0) | 64(4.8) | 9(5.1) | 9.5; 0.050 |
| Glomerulonephritis, n(%) | 160(3.4) | 51(15.0) | 36(3.5) | 41(2.2) | 26(1.9) | 6(3.4) | 156.1; <0.001 |
| Single kidney, n(%) | 226(4.8) | 18(5.3) | 69(6.8) | 77(4.2) | 53(4.0) | 9(5.1) | 12.8; 0.012 |
| Unknown nephropathy, n(%) | 233(4.9) | 23(6.8) | 59(5.8) | 82(4.4) | 60(4.5) | 8(4.5) | 5.7; 0.221 |
| Rare nephropathies, n(%) | 64(1.4) | 8(2.3) | 16(1.6) | 16(0.9) | 20(1.5) | 4(2.3) | 7.5; 0.113 |
| Diabetes, n(%) | 1569(33.3) | 89(26.2) | 293(18.9) | 626(33.9) | 506(37.8) | 54(30.7) | 29.5; <0.001 |
| Cardiovascular comorbidities, n(%) | 988(21.0) | 31(9.1) | 134(13.2) | 382(20.7) | 388(29.0) | 52(29.7) | 125.8; <0.001 |
| COPD, n(%) | 239(5.1) | 3(0.9) | 37(3.6) | 103(5.6) | 86(6.4) | 9(5.1) | 22.9; <0.001 |
| Tumors, n(%) | 321(6.8) | 16(4.7) | 64(6.3) | 125(6.8) | 99(7.4) | 17(9.7) | 5.8; 0.213 |
| Liver disease, n(%) | 43(0.9) | 1(0.3) | 11(1.1) | 12(0.6) | 18(1.3) | 1(0.6) | 6.2; 0.188 |
| Dementia, n(%) | 106(2.2) | 2(0.6) | 14(1.4) | 42(2.3) | 42(3.1) | 6(3.4) | 13.7; 0.008 |
| BMI (kg/m^2^), mean±sd | 28.0±4.8 | 27.5±4.4 | 28.1±4.4 | 28.3±4.9 | 27.9±5.1 | 26.1±4.8 | 43.7; <0.001* (5<all) |
| Immigrants, n(%) | 176(3.7) | 27(7.9) | 26(2.6) | 70(3.8) | 46(3.4) | 7(4.0) | 20.9; <0.001 |
| Patients not regularly followed up by nephrologists, n(%) | 1576(33.4) | 194(57.1) | 488(48.1) | 611(33.1) | 269(20.1) | 13(7.4) | 344.9; <0.001 |

Note: * Kruskal-Wallis test and Conover post-hoc comparison with Holm adjustment.

**Table C. Characteristics of the study cohort by province**

|  | Overall sample (n=4716) | Forlì-Cesena (n=1827, 38.7%) | Ravenna (n=852, 18.1%) | Rimini (n=2037, 43.2%) | χ^2^ test; p-value |
| --- | --- | --- | --- | --- | --- |
| Age at 1.01.2020, mean±sd | 76.2±11.6 | 75.3±12.2 | 74.2±12.5 | 77.9±10.4 | 71.2; <0.001* (RN>RA and FC) |
| Males, n(%) | 3086 (65.4) | 1202 (65.8) | 597 (70.1) | 1287 (63.2) | 12.8; 0.002 |
| eGFR at last visit (ml/min/1.73m^2^), mean±sd | 38.1±15.8 | 42.1±17.6 | 34.7±16.6 | 35.8±12.8 | 173.7; <0.001* (FC>RN>RA) |
| Hypertensive nephropathy, n(%) | 3274 (69.4) | 1100 (60.2) | 563 (66.1) | 1611 (79.1) | 167.2; <0.001 |
| Diabetic nephropathy, n(%) | 432 (9.2) | 212 (11.6) | 87 (10.2) | 133 (6.5) | 31.2; <0.001 |
| Polycystic kidney, n(%) | 98 (2.1) | 31 (1.7) | 25 (2.9) | 42 (2.1) | 4.4; 0.112 |
| Pyelonephritis, n(%) | 229 (4.9) | 123 (6.7) | 30 (3.5) | 76 (3.7) | 22.8; <0.001 |
| Glomerulonephritis, n(%) | 160 (3.4) | 76 (4.2) | 39 (4.6) | 45 (2.2) | 15.6; <0.001 |
| Single kidney, n(%) | 226 (4.8) | 114 (6.2) | 70 (8.2) | 42 (2.1) | 63.6; <0.001 |
| Unknown nephropathy, n(%) | 233 (4.9) | 139 (7.6) | 28 (3.3) | 66 (3.2) | 45.2; <0.001 |
| Rare nephropathies, n(%) | 64 (1.4) | 32 (1.7) | 10 (1.2) | 22 (1.1) | 3.5; 0.173 |
| Diabetes, n(%) | 1569(33.3) | 641(35.1) | 288(33.8) | 640(31.4) | 6.0; 0.051 |
| Cardiovascular comorbidities, n(%) | 988(21.0) | 360(19.7) | 181(21.3) | 447(22.0) | 3.1; 0.216 |
| COPD, n(%) | 239(5.1) | 73(4.0) | 52(6.1) | 114(5.6) | 7.5; 0.023 |
| Tumors, n(%) | 321(6.8) | 117(6.4) | 67(7.9) | 137(6.7) | 2.0; 0.362 |
| Liver disease, n(%) | 43(0.9) | 15(0.8) | 10(1.2) | 18(0.9) | 0.8; 0.657 |
| Dementia, n(%) | 106(2.2) | 39(2.1) | 17(2.0) | 50(2.5) | 0.7; 0.686 |
| BMI (kg/m^2^), mean±sd | 28.0±4.8 | 28.3±4.7 | 27.6±4.8 | 27.9±5.0 | 22.6; <0.001* (FC>RA and RN) |
| Immigrants, n(%) | 176 (3.7) | 51 (2.8) | 54 (6.3) | 71 (3.5) | 20.9; <0.001 |
| Patients not regularly followed up by nephrologists , n(%) | 1576 (33.4) | 669 (36.6) | 222 (26.1) | 685 (33.6) | 29.2; <0.001 |

Note: * Kruskal-Wallis test and Conover post-hoc comparison with Holm adjustment.

**Table D: COVID-19 mortality in CKD patients by demographic and clinical characteristics.**

|  | **N. of cases (n=193)** | **N. of deaths (n=86)** | **Log-rank test (z; p-value)** |
| --- | --- | --- | --- |
| Age at January 1st, 2020 |  |  | 10.41; 0.034 |
| <60 years | 4 | 0 |  |
| 60-69 years | 16 | 3(18.7%) |  |
| 70-79 years | 57 | 22(38.6%) |  |
| 80-89 years | 88 | 46(52.3%) |  |
| ≥90 years | 28 | 15(53.6%) |  |
| Males | 123 | 54(43.9%) | 0.36; 0.551 |
| Immigrants | 6 | 0 | 3.58; 0.058 |
| Provinces |  |  | 0.01; 0.997 |
| Ravenna, n(%) | 22 | 10(45.5%) |  |
| Forlì-Cesena, n(%) | 62 | 29(46.8%) |  |
| Rimini, n(%) | 109 | 47(43.1%) |  |
| Period of onset |  |  | 19.5; 0.003 |
| 19.01.2020 – 7.03.2020 | 29 | 15(51.7%) |  |
| 08.03.2020 – 21.03.2020 | 28 | 20(71.4%) |  |
| 22.03.2020 – 04.04.2020 | 28 | 14(50.0%) |  |
| 05.04.2020 – 18.04.2020 | 28 | 14(50.0%) |  |
| 19.04.2020 – 02.05.2020 | 24 | 10(41.7%) |  |
| 03.05.2020 – 30.05.2020 | 42 | 12(28.6%) |  |
| 31.05.2020 – 31.07.2020 | 14 | 1(7.1%) |  |
| CKD-EPI stage |  |  | 8.44; 0.038 |
| 1-2 (eGFR>60 mL/min) | 4 | 1(25.0%) |  |
| 3a (eGFR 45-60 mL/min) | 25 | 7(28.0%) |  |
| 3b (eGFR 30-45 mL/min) | 72 | 29(40.3%) |  |
| 4-5 (eGFR <30 mL/min) | 91 | 49(53.9%) |  |
| BMI (kg/m^2^) |  |  | 6.35; 0.096 |
| <25 | 51 | 29(56.9%) |  |
| 25-29.99 | 68 | 24(34.8%) |  |
| 30-34.99 | 47 | 23(48.9%) |  |
| ≥35 | 18 | 8(42.1%) |  |
| Hypertensive nephropathy | 150 | 70(46.7%) | 2.18; 0.140 |
| Diabetes | 69 | 31(44.9%) | 0.00; 0.945 |
| Cardiovascular comorbidities | 77 | 40(51.9%) | 1.94; 0.164 |
| COPD | 26 | 12(46.1%) | 0.01; 0.926 |
| Tumors | 21 | 11(52.4%) | 0.24; 0.621 |
| Dementia | 10 | 8(80.0%) | 10.7; 0.001 |

**Table E: Characteristics of patients included in the multiple regression analysis of COVID-19 in-hospital mortality.**

|  | **PIRP (n=187)** | **Non-PIRP (n=5505)** | **Test; p-value** |
| --- | --- | --- | --- |
| Age, mean±SD | 81.5±8.3 | 69.6±19.3 | -8.4; <0.001 |
| Males, n(%) | 121(64.7) | 2914(52.9) | 10.1; 0.002 |
| Areas of AUSL Romagna, n(%) |  |  | 20.6; <0.001 |
| Ravenna | 22(11.8) | 1453(26.4) |  |
| Forlì-Cesena | 60(32.1) | 1572(28.6) |  |
| Rimini | 105(56.1) | 2480(45.0) |  |
| Diabetes, n(%) | 23(12.3) | 304(5.5) | 15.3; <0.001 |
| Cardiovascular comorbidities, n(%) | 58(31.0) | 684(12.4) | 55.1; <0.001 |
| COPD, n(%) | 24(12.8) | 277(5.0) | 22.0; <0.001 |
| Tumors, n(%) | 16(8.6) | 381(6.9) | 0.7; 0.388 |
| Liver disease, n(%) | 2(1.1) | 70(1.3) | 0.1; 0.808 |
| Dementia, n(%) | 7(3.7) | 188(3.4) | 0.1; 0.808 |
